# Bridging the Treatment Gap in Kidney Failure Through Transplantation—A Model for Africa and Beyond

**DOI:** 10.1097/TXD.0000000000001915

**Published:** 2026-03-17

**Authors:** Elmi Muller

**Affiliations:** 1 Department of Surgery, Faculty of Medicine and Health Sciences, Stellenbosch University, Tygerberg, Cape Town, South Africa.

In many parts of the world, the words “end-stage kidney disease” are effectively a death sentence. Not because of the severity of the disease alone but because of the systemic failure to provide equitable access to life-saving treatments. In this issue of *Transplantation Direct*, the latest study by Manning et al^1^ reports their findings on access to kidney replacement therapy (KRT) and outcomes among an uninsured cohort who access the public sector in the Eastern Cape province of South Africa. The findings are confronting: they provide a sobering account of health system limitations for patients in kidney failure, yet provide a powerful case for the transformative potential of kidney transplantation. It should serve as a blueprint for low- and middle-income countries (LMICs) facing similar challenges.

The Eastern Cape is one of the poorest provinces in South Africa, marked by vast geographic distances, high unemployment, and a fragile public health infrastructure, with only 7% of the population having medical insurance. The first of many important findings, Manning et al showed that only 20% of public sector patients referred with kidney failure receive any form of treatment. The remaining 80% are, in effect, sentenced to die:often young, often economically active, and often entirely outside the purview of health system metrics. This is not just a tragedy; it is a policy failure.

What sets this study apart is not only its rigorous methodology and comprehensive data set, but also its longitudinal perspective: patients were followed from kidney failure diagnosis to outcome from 2012 to 2020. The application of the competing risks approach alongside Kaplan-Meier survival estimates provides novel and nuanced insights into the progression from dialysis initiation to transplantation, death, or graft failure. The survival outcomes are stark. Among those who remained on dialysis, the 5-y survival was 61%. Among those transplanted, it was 100%. In other words, the choice between dialysis and transplantation is not merely clinical: it is existential.

Important to note is that only one in five patients accepted onto dialysis in this cohort ever received a transplant. This is despite favorable socioeconomic and clinical profiles among patients prioritized for KRT (due to transplantation eligibility). The bottlenecks, whether due to resource constraints, systemic inefficiencies, or limitations in regional transplant capacity, speak volumes about the structural inequities that underlie KRT provision in low- and middle-income countries.

These findings have significant implications. First, they force us to reexamine the metrics we use to estimate disease burden. National renal registries, including that of South Africa, typically reflect only treated kidney failure. This article demonstrates how much remains uncounted: those who never make it to dialysis, who die unrecorded and unacknowledged. Second, the data underscore the need to prioritize transplantation over dialysis as a long-term solution in the public sector. Dialysis, although essential, is a costly and often unsustainable palliative measure. Transplantation, by contrast, offers superior survival and quality-of-life outcomes and ultimately proves more cost-effective.

This insight aligns with broader international discourse. In a 2025 article published in *Transplantation*, the global summit on transplantation under the auspices of the Spanish Presidency of the EU emphasized that transplantation is not only a clinical intervention but also a strategic tool to strengthen health systems.^2^ Transplantation requires robust infrastructure, ethical governance, trained personnel, and efficient referral pathways. When these systems are in place, they elevate care across disciplines.

Moreover, prioritizing transplantation contributes to sustainable health development and fulfills the objectives of multiple UN Sustainable Development Goals (SDGs). These include SDG 3 (Good Health and Well-being), SDG 8 (Decent Work and Economic Growth), and SDG 10 (Reduced Inequality). By reducing the economic burden of chronic disease and promoting equity in access to care, transplantation is not a luxury but a necessity.

South Africa’s 2-tiered health system presents an illustrative case. Although private-sector patients with kidney failure often access dialysis and transplantation with relative ease, the public sector relies on rationing and strict eligibility criteria. The result is a massive treatment gap between insured and uninsured populations. The Eastern Cape data show what happens when this policy intersects with poverty: treatable disease becomes terminal.

Yet, what is perhaps most compelling in this work is the hope it offers. The Eastern Cape programme, though small and underresourced, demonstrates excellent transplant outcomes. Graft survival at 5 y exceeds 95%. Mortality posttransplant is negligible. These are not statistics from a flagship academic center: they are from one of South Africa’s most underfunded provinces. If this is possible here, it is possible anywhere.

To translate this model across Africa, we need more than clinical evidence. We need political will. Governments must recognize that dialysis expansion alone will not solve the growing burden of kidney disease. Investment in regional transplant capacity, including surgical infrastructure, donor systems, immunological support, and follow-up care, must be prioritized. The current default to dialysis reflects market dynamics more than patient outcomes; dialysis is a global industry, transplantation a public good.

We must also shift the narrative. Transplantation is not a “last resort” for the few, but a first-line therapy for the many. It is a platform for equity, a catalyst for system improvement, and a humane response to a growing epidemic. This study makes a compelling case: if the Eastern Cape can do it, so can the rest of Africa.

The time to act is now. We can no longer accept untreated kidney failure as an inevitability. We must recognize it for what it is: a preventable, system-induced death. And we must commit to a future in which transplantation is not a privilege, but a right.

E.M. participated in writing the article.
